# Protocol for an Economic Evaluation of the Quitlink Randomized Controlled Trial for Accessible Smoking Cessation Support for People With Severe Mental Illness

**DOI:** 10.3389/fpsyt.2019.00618

**Published:** 2019-09-03

**Authors:** Rohan Sweeney, Marj Moodie, Amanda L. Baker, Ron Borland, David Castle, Catherine Segan, Alyna Turner, John Attia, Peter J. Kelly, Lisa Brophy, Billie Bonevski, Jill M. Williams, Donita Baird, Sarah L. White, Kristen McCarter

**Affiliations:** ^1^Centre for Health Economics, Monash Business School, Monash University, Melbourne, VIC, Australia; ^2^Deakin Health Economics, Centre for Population Health Research, Deakin University, Geelong, VIC, Australia; ^3^Faculty of Health and Medicine, University of Newcastle, Newcastle, NSW, Australia; ^4^The Cancer Council Victoria, Melbourne, VIC, Australia; ^5^School of Psychological Sciences, University of Melbourne, Melbourne, VIC, Australian; ^6^Department of Psychiatry, University of Melbourne, Melbourne, VIC, Australia; ^7^Department of Psychiatry, St Vincent’s Hospital Melbourne, Fitzroy, VIC, Australia; ^8^Melbourne School of Population and Global Health, University of Melbourne, Melbourne, VIC, Australia; ^9^IMPACT Strategic Research Centre, School of Medicine, Deakin University, Geelong, VIC, Australia; ^10^Barwon Health, Geelong, VIC, Australia; ^11^Illawarra Institute for Mental Health, School of Psychology, University of Wollongong, Wollongong, NSW, Australia; ^12^Mind Australia, Melbourne, VIC, Australia; ^13^Division of Addiction Psychiatry, Rutgers Robert Wood Johnson Medical School and Rutgers Cancer Institute of New Jersey, New Brunswick, NJ, United States

**Keywords:** smoking, smoking cessation, mental illness, quitline, peer worker, economic evaluation, cost-effectiveness

## Abstract

**Introduction:** Smoking is a major cause of disease burden and reduced quality of life for people with severe mental illness (SMI). It places significant resource pressure on health systems and financial stress on smokers with SMI (SSMI). Telephone-based smoking cessation interventions have been shown to be cost effective in general populations. However, evidence suggests that SSMI are less likely to be referred to quitlines, and little is known about the effectiveness and cost effectiveness of such interventions that specifically target SSMI. The Quitlink randomized controlled trial for accessible smoking cessation support for SSMI aims to bridge this gap. This paper describes the protocol for evaluating the cost effectiveness of Quitlink.

**Methods:** Quitlink will be implemented in the Australian setting, utilizing the existing mental health peer workforce to link SSMI to a tailored quitline service. The effectiveness of Quitlink will be evaluated in a clustered randomized controlled trial. A cost-effectiveness evaluation will be conducted alongside the Quitlink clustered randomized controlled trial (RCT) with incremental cost-effectiveness ratios (ICERs) calculated for the cost (AUD) per successful quit and quality adjusted life year (QALY) gained at 8 months compared with usual care from both health care system and limited societal perspectives. Financial implications for study participants will also be investigated. A modeled cost-effectiveness analysis will also be conducted to estimate future costs and benefits associated with any treatment effect observed during the trial. Results will be extrapolated to estimate the cost effectiveness of rolling out Quitlink nationally. Sensitivity analyses will be undertaken to assess the impact on results from plausible variations in all modeled variables.

**Discussion:** SSMI require additional support to quit. Quitlink utilizes existing peer worker and quitline workforces and tailors quitline support specifically to provide that increased cessation support. Given Quitlink engages these existing skilled workforces, it is hypothesized that, if found to be effective, it will also be found to be both cost effective and scalable. This protocol describes the economic evaluation of Quitlink that will assess these hypotheses.

**Ethics and dissemination:** Full ethics clearances have been received for the methods described below from the University of Newcastle (Australia) Human Research Ethics Committee (H-2018-0192) and St Vincent’s Hospital, Melbourne (HREC/18/SVHM/154). The trial has been registered with the Australian and New Zealand Clinical Trials Registry (ACTRN12619000244101). Participant consent is sought both to participate in the study and to have the study data linked to routine health administrative data on publicly subsidized health service and pharmaceutical use, specifically the Medicare Benefits and Pharmaceutical Benefits Schemes (MBS/PBS). Trial findings (including economic evaluation) will be published in peer reviewed journals and presented at international conferences. Collected data and analyses will be made available in accordance with journal policies and study ethics approvals. Results will be presented to relevant government authorities with an interest in cost effectiveness of these types of interventions.

## Introduction

While smoking rates have declined in many countries, the rate of decline among people living with severe and enduring mental illness (SMI) has been significantly slower (1, 2). For example, in the USA, over the period 2004–2011, smoking among individuals with no mental illness declined from 19.5% to 15.6% (*p* < 0.001), compared with 28.8% to 27.0% (*p* = 0.006) among individuals living with mental illness (2). Smoking rates in people living with SMI have been found to be around double the general population and up to four times higher for those living with bipolar disorder or schizophrenia (1, 3, 4). Smoking increases the risk of a number of tobacco-related illnesses, including lung, throat and bowel cancers, stroke, chronic obstructive pulmonary disease (COPD), and myocardial infarction (5). Consequently, smoking is the leading cause of preventable death among people living with SMI—significantly shortening their life expectancy compared to the general population and accounting for almost half of all smoking-related deaths (4, 6–9).

Smoking-related conditions also cause significant morbidity and reduce the quality of life of affected people, with or without the presence of SMI (5, 10). Exacerbating this for people living with SMI, smoking has been associated with increased psychiatric symptoms and hospitalizations, as well as a requirement for higher psychiatric medication dosages because smoking accelerates the metabolism of some antidepressant and antipsychotic medications (11, 12).

Data on the economic burden associated with smoking in people with SMI are limited, but evidence suggests that it is significant. In Australia in 2007, it was estimated, that when compared to smokers without mental illness, the additional cost of health care, lost productivity, carer costs, cigarette expenditure, and other costs associated with observed heavier levels of smoking among ∼1.25 million smokers with mental illness (not just SMI), was around AUD3.5 billion annually (or about AUD4.5 billion in 2018^1^) (13). This is in addition to expected costs if smokers with mental illness smoked at similar levels to smokers with no mental illness—the main cost drivers being productivity losses (63%), health costs (12%), and cigarette expenditure (12%) (13). In the 2009/2010 UK financial year, it was estimated that the costs associated with smoking-related health care treatment, work-related absenteeism, and premature mortality among people with SMI was £2.3 billion (or about £3 billion in 2018^2^) (14).

Numerous smoking cessation strategies have been shown to be both effective and cost effective in the general population (15, 16). However, smokers with severe mental illness (SSMI) report lower cessation rates, in part attributable to higher levels of nicotine dependence, and they are likely to benefit from more intensive or extended interventions tailored to their needs (17). SSMI also report a lack of encouragement to quit by health professionals, who often mistakenly believe that people with mental illness are not interested in quitting and that it will interfere with their mental health recovery (12, 18).

Given the significant disease burden caused by smoking among people with SMI, improving access to smoking cessation interventions—and ensuring they are effective for SSMI—is a vital health priority for this target group. Telephone-based smoking cessation counseling services (such as quitlines) are helpful for many smokers, but SSMI are infrequently referred to such services by mental health practitioners as it is uncommon for smoking cessation to be included in mental health planning (19). This has led to the development of Quitlink—a randomized controlled trial (RCT) of peer worker facilitated quitline support for smokers with mental health problems, implemented in an Australian setting (20). It aims to coordinate and enhance the services of Quitline Victoria and engage mental health peer workers to bridge the persistent gap between mental health services and Quitline. The primary aim of the intervention is to help SSMI quit smoking. Secondary aims include assessment of the extent to which Quitlink improves health-related quality of life (HRQoL) and reduces the burden on the health care system in both the short and longer terms.

Examining the cost effectiveness of proven or potentially effective interventions is increasingly important for public sector funding decisions and priority setting (21, 22). Telephone-based counseling interventions with or without complementary nicotine replacement therapy (NRT) can be a relatively cost-effective way to achieve smoking abstinence in general populations in both upper and lower income country settings (16). Furthermore, modeling suggests such interventions may even be cost saving from a health care system perspective due to cost offsets resulting from prevented health costs in the future (23–26).

While it has been well established that telephone-based counseling interventions (with or without NRT) can be a very cost-effective strategy for improving health and extending lives (15, 16), there is little evidence regarding the cost effectiveness of any smoking cessation strategies specifically targeting SSMI (27). Barnett et al. (28) compared a cessation program (including psychological counseling, NRT, and bupropion) given in an outpatient care setting in the USA for smokers with depression measured against a brief care comparator. After 18 months, the intervention group had a 5.5% increased chance of ceasing smoking (*p* < 0.05) at a cost of USD11,496 per successful quit and USD9,580 per life year gained, concluding that it was a relatively cost-effective intervention in the short run. In a more recent RCT, Barnett et al. (29) found a stage-based intervention (including computer-based assessment, regular feedback, face to face sessions, and up to 10 weeks of NRT) initiated with people during a psychiatric hospitalization, was highly cost effective. The intervention achieved around a 12 percentage point increase in smoking abstinence after 18 months compared with usual care [18.8% abstinence in the intervention arm versus 6.8% abstinence in usual care (*p* < 0.05)] at an estimated USD428 per additional quality adjusted life year (QALY) gained when modeled over the life course of participants. Rejas-Gutiérrez et al. (30) constructed a model to estimate the budgetary impact for the Spanish health care system from funding varenicline, bupropion, and NRT combined with medical follow-up and counseling for people with a major depressive disorder. They estimated that the cost of funding such interventions (€25.3 million) was offset by health costs avoided (€26.5 million) after 5 years, suggesting that cost offsets for the health care system might increase over a longer time period (30).

The Quitlink intervention will utilize existing and skilled Quitline and mental health peer workers. The peer workforce is developing in Australia and internationally, working alongside clinical staff to provide support based on shared lived experience of mental illness and recovery (31). In Australia, quitlines are government-funded services providing smoking cessation counseling across each state and territory. In addition, in the Australian setting, some of the medications to aid smoking cessation are currently subsidized. The presence of these funded health resources suggests that the additional resources required to implement Quitlink would be relatively modest. It is hypothesized then that, if effective, it is likely to be a highly cost-effective intervention, which can feasibly be scaled up beyond the trial setting. Such *a priori* expectations make the case for a rigorous economic evaluation to be conducted alongside the Quitlink RCT. This paper presents a protocol for the economic evaluation of the Quitlink intervention to address the following research question:

From the Australian health care system and limited societal perspectives, what is the cost effectiveness of the Quitlink intervention to increase smoking cessation and QALYs among people living with SMI when compared with usual care?

## The Quitlink Trial

### Study Design

Quitlink is a cluster RCT, the design of which is described in detail in Baker et al. (20). In brief, a multicenter prospective, randomized, open, blinded endpoint design will be utilized to compare Quitlink against usual smoking care in helping SSMI to quit smoking. The trial aims to recruit 382 participants with SMI from participating residential and nonresidential, hospital, and community-based mental health services in Victoria, Australia. The trial will entail cluster randomization: where individuals are part of a short- or long-term residential rehabilitation program, that residential program will be considered a cluster. Where individuals are not part of a residential rehabilitation program, they will be randomized individually, i.e., a cluster of 1. Participants randomized to the intervention group will receive the full Quitlink intervention as described below. All participants will undergo follow-up at 2, 5, and 8 months postbaseline. The main outcomes are described below in the section *Identification, Measurement, and Valuation of Outcomes* and described in detail in Baker et al. (20). A qualitative study will investigate the experience of participants with a focus on further enhancing engagement with the intervention. Full ethics approval for the methods described here and in Baker et al. (20) was obtained from the University of Newcastle (H-2018-0192) and St Vincent’s Hospital, Melbourne (HREC/18/SVHM/154).

### Screening, Randomization, and the Usual Care Control Group

Potential participants will be engaged and screened for eligibility by a trained mental health peer worker at specialist mental health services [see Baker et al. (20) for further details]. For eligible persons, upon provision of informed consent, a baseline assessment will be undertaken. Following this, participants will receive a brief smoking cessation intervention consisting of brief advice and provision of Quit Victoria written materials that include the Quitline telephone number.

After the provision of this brief smoking cessation intervention, participants will be randomly allocated to the control group or the Quitlink intervention group. The control group will continue with usual care in relation to smoking cessation support, as provided by their health care team, that is, no further intervention will be provided by the research team.

### The Intervention

Following randomization, those allocated to the intervention will be referred to an enhanced quitline call-back service for SSMI and have the option of receiving up to 8 weeks of NRT (patches, complemented by an oral form of NRT, to be used as per pack guidelines). Quitline will proactively contact the participant to offer up to 8 weeks of telephone smoking cessation counseling with a dedicated counselor, which will include monitoring of mental health symptoms, nicotine withdrawal symptoms, and medication side effects, as well as mood management strategies that aid cessation.

## Methods and Analysis

### Economic Evaluation Overview

A cost-effectiveness evaluation will be conducted with incremental cost-effectiveness ratios (ICER) calculated for the cost (AUD) per person who quits and QALYs gained compared with usual care from both health care system and limited societal perspectives. Cost effectiveness will be estimated at 8 months postrandomization (trial-based evaluation with costs and outcomes as per the trial). Given that most of the anticipated benefits associated with smoking cessation will occur well beyond the trial period (15, 16, 24), downstream costs and benefits will be estimated *via* a modeled economic evaluation. Health care system costs and health and QALY benefits will be estimated over the life course of study participants and extrapolated to estimate the cost effectiveness of rolling out Quitlink nationally.

The health care system perspective will be of most relevance to agencies that are likely to fund scaleup beyond the trial setting. Data on important personal out-of-pocket impacts of Quitlink will also be collected and incorporated with the health care system data to construct the limited societal perspective analysis. In the Australian setting, cigarettes are highly taxed (to reduce smoking) and are among the most expensive in the world, while the population of people with SMI is generally financially disadvantaged and often marginalized economically (32, 33). This makes it important to also assess any consequent financial impacts on study participants as a result of receiving the Quitlink intervention.

Future costs and benefits will be discounted using an annual discount rate of 3% in the base-case. Furthermore, annual discount rates of 0 and 5% will be applied in sensitivity analysis to facilitate comparison with results from other economic evaluations of preventive health interventions, including smoking cessation interventions in people with SMI (25, 29, 34). To further aid decision-makers, cost-effectiveness findings will be presented alongside descriptive assessments of the acceptability to stakeholders, feasibility of scaleup, sustainability, and equity implications of Quitlink implementation to be assessed by the research team in consultation with participating organizations (34, 35).

### Trial-Based Economic Evaluation

#### Identification, Measurement, and Valuation of Outcomes

The clinical and HRQoL outcomes detailed below will be collected as part of participant assessments conducted at baseline, 2 months (= end of treatment), 5 months (= 3 months posttreatment), and 8 months (= 6 months posttreatment).

##### Health and Health-Related Behavioral Outcomes

The primary health outcome will be successful quits at 8 months postrandomization. A successful quit is defined as 6 months sustained abstinence, with no relapse of 7 or more days of continuous smoking, and no reported smoking in the past week with biochemical verification). Self-reported cigarette consumption will also be measured and for the purposes of the economic evaluation, used to assess changes in out of pocket expenditure associated with Quitlink.

##### Health-Related Quality of Life

Despite common beliefs that smoking cessation might worsen the mental health symptoms of smokers, some studies indicate that smoking cessation leads to no worsening and possibly improvement in mental health and psychological-related quality of life (36). It is also plausible that mood and mental well-being symptoms may change over time, e.g., deteriorate in the short term while quitting (e.g., first few weeks), and improve after that (e.g., months after successfully quitting) (36). To explore this, HRQoL data will be collected using the Assessment of Quality of Life-8 Dimension (AQoL-8D) instrument at baseline and follow-up observations at 2, 5, and 8 months. The AQoL-8D is a preference-based HRQoL instrument which enables calculation of QALYs experienced across the two study arms. Data from all time points will be plotted for both arms, and the difference in areas under the respective curves will be calculated. While the majority of benefit of this preventive intervention are expected in the future and *a priori* expectations of measurable change in HRQoL during the trial period are modest, among preference-based HRQoL instruments, the AQoL-8D is considered relatively sensitive to changes in psychosocial dimensions of HRQoL (while also capturing important changes in other dimensions of HRQoL) (37). This means that it will be more likely to identify smaller changes in mental-health-related quality of life than other preference-based instruments.

##### Financial Stress

Respondents will be asked a short module of questions relating to their financial stress at baseline and follow-up observations (38). For example, have they foregone meals; asked for financial help; or been unable to pay electricity, gas, or telephone bills because of a lack of money (see **online Appendix** to view questions)? This will provide further evidence for decision-makers and mental health and smoking program organizations regarding potential financial impacts of Quitlink on this financially disadvantaged population (32).

#### Identification and Measurement of Costs

**Table 1** summarizes the costs included and the data collection strategy from both a health sector and societal perspective. Costs included from the health care system perspective will include direct intervention costs (e.g., opportunity cost of Quitline and peer worker staff, telephone calls, NRT) for both Quitlink and usual care, as well as drug and health service utilization costs. Pathway analysis will be undertaken to ensure all relevant costs are identified. These data will be collected from project administrative records, respondent surveys (baseline, 2, 5, and 8 months) and with participant consent, linked data on their service and prescription medication use from the Australian Government subsidized Medicare (MBS) and Pharmaceutical Benefits (PBS) schemes, which are predominantly out-of-hospital resource use.

**Table 1:** Costs included in trial-based cost-effectiveness analyses.

Cost category	Costs	Perspective	Collection strategy
Direct intervention costs	Costs associated with training of peer workers and Quitline staff, including personnel time (facilitators and participants), venue/catering, printing/stationery.	HS & S	Project administrative records.
	Personnel time for intervention delivery: Quitline and peer worker support time spent per study participant in both Quitlink and usual care arms. Costs of telephone calls. Program management time. On-costs will be included.	HS & S	Project administrative records and administrative data provided by participating organizations.
Health service utilization	Hospitalizations (including length of stay) and other intensive health services, including ED and community care units (CCUs) and prevention and recovery care services (PARCS).	HS & S	Respondent surveys.
	Community-based (noninpatient) government subsidized health (including mental health) services.	HS & S	Linkage to Australian Department of Human Services data on Medicare and PBS use. Literature review.
	Allied health services (nonsubsidized) including (non-Quitline) counseling, acupuncture, hypnotherapy, group therapies.	S	Respondent surveys.
Nicotine replacement therapies and other quitting aids	e.g., patches, gum, lozenges, inhalator, sprays, e-cigarettes.	HS & S	Respondent surveys.
Medicines	Including varenicline, bupropion, psychotropic medicines.	HS & S	Australian Department of Human Services data on Medicare and PBS use, literature review.
Cigarettes	Cost of cigarette purchases.	S	Respondent surveys.
Productivity losses and gains	Absenteeism from paid and unpaid work (e.g., volunteering, study, caring). Potential increases in employment.	S	Respondent surveys. Project records on session numbers and duration.

HS, Health care system; S, societal; ED, emergency department; PBS, Pharmaceutical Benefits Scheme.

Data on important out-of-pocket impacts of Quitlink will also be collected and incorporated with the health care system data to estimate costs from a limited societal perspective. These costs will include out-of-pocket co-payments associated with drug and health service utilization, expenditure on cigarettes, and cessation aids purchased in addition to those provided as part of the intervention, as well as time costs and productivity losses associated with absenteeism from paid and unpaid work and productive activities (see **online Appendix** to view questions).

Where data relies on respondent recall, for example, number of allied health visits or cigarettes smoked, the recall period will be deliberately kept relatively short (1 month and 1 week, respectively). Recall bias may remain an issue though, so the potential impact of this will be tested in sensitivity analyses (39). In general, a simple extrapolation rule will be followed where reported rates are applied for the full period since previous follow-up, where appropriately justified.

One-off costs for products which could be used in other settings, such as costs of developing the training and intervention materials, will be excluded. The costs and health implications from passive smoking will also be excluded.

#### Valuing Costs

All resource use will be costed using nationally published reference costs or market prices where appropriate. Personnel time (paid, unpaid, volunteer time) will be costed using opportunity cost principles, where volunteer/leisure time will be valued at 25% of appropriate average wage rates (34). Resource use of nonhealth sector goods and services will be valued at market prices and be informed by best available evidence from Australian-based studies. Where relevant, health resources will be costed as per the Manual of Resource Items for use in submissions to the Commonwealth of Australia’s Pharmaceutical Benefits Advisory Committee (40). Health care cost information will also be drawn from the Australian Institute of Health and Welfare (AIHW) health care cost data. All costs will be inflated to current Australian dollars for the year of study completion (2022) using the all-items Consumer Price Index from the Australian Bureau of Statistics.

### Modeling Long-Term Cost Effectiveness

A decision analytic Markov model will be developed using *TreeAge* software to estimate the future benefits and cost savings arising from any increase in successful quits observed in the Quitlink arm. We will adapt and update the smoking cessation model developed with an Australian context by Hurley et al. (41). The model projects the future smoking status of the population where smoking status impacts on the risk of experiencing (progressing into the following health states)—myocardial infarction (MI), stroke, COPD, or lung cancer. These four health states are known to have the largest disease, mortality, and health cost burden among smokers (41). For simplicity, the model does not include the potential for comorbid health states where a person may have more than one of these four states concurrently. Death following transition into one of these diagnosed health states can be caused by that condition or any other cause. “Healthy” smokers and ex-smokers can also die from other causes without experiencing these health states. This approach is intentionally conservative (i.e., it likely underestimates the benefits of quitting) and has been taken in a number of smoking models (24).

**Figure 1:**
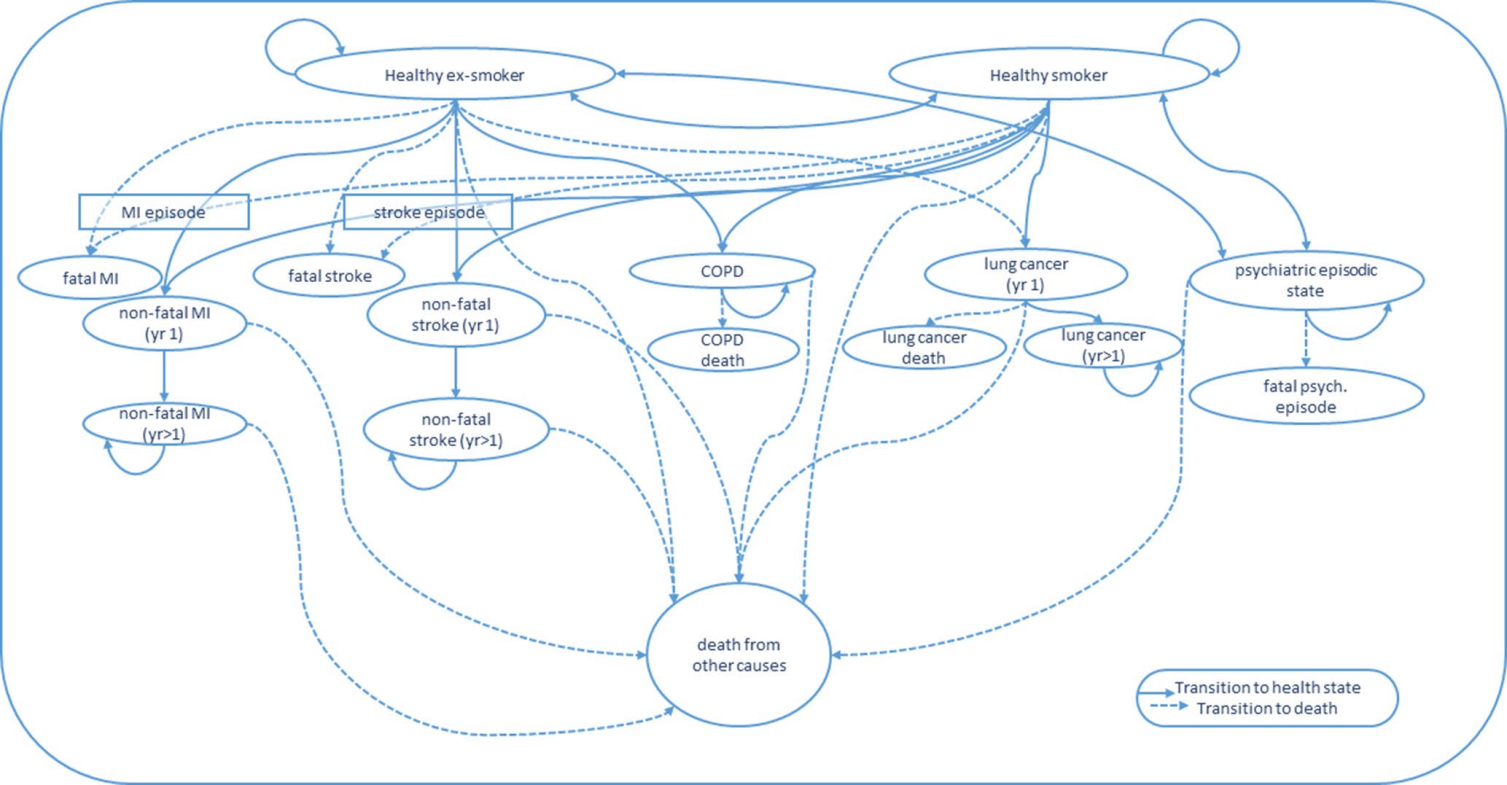
State transitions for Markov Model. COPD, chronic obstructive pulmonary disease; MI, myocardial infarction; yr 1, first year in a given health state; yr > 1, subsequent years lived in a given health state.

We plan to extend the Hurley and Matthews (41) model. **Figure 1** depicts the potential health states that the modeled Quitlink cohort will face over repeat model annual cycles. Given smokers with SMI (compared to ex-smokers with SMI) face increased risk of hospitalization for a psychiatric episode, a psychiatric episodic health state will be added to the model to capture the costs related to hospitalizations and the impact on QALYs (12). QALY weights for the psychiatric episodic health state will be obtained where possible, from the literature or by expert opinion, guided by the AQoL-8D questionnaire. In the model, we will also consider that smoking cessation may reduce suicide risk—attempts and, more rarely, deaths (42, 43). Uncertainty remains around this mechanism of action. However, the known links between smoking and reduced effectiveness of antipsychotic medication, and between smoking cessation and mood improvement, suggest that any increased smoking cessation achieved by Quitlink may plausibly reduce suicide attempts and deaths—especially if the program was scaled up. A review of the literature of the causal link between smoking and suicidality will be undertaken at the end of the trial period to determine the strength of evidence and suitability of including a suicidal health state in the Markov model and suicide as an additional smoking-related cause of death.

The trial cohort at the end of the trial follow-up will enter the Markov model as either a healthy smoker or healthy ex-smoker (i.e., successful quitter), where “healthy” means they have not had a stroke, MI, or developed COPD or lung cancer. Their commencement QALY weight in the model will be their final observed QALY weight from the trial (i.e., 8-month follow-up). Individuals will be modeled through annual cycles. In the first cycle, people have a probability of either remaining a healthy smoker or ex-smoker, relapsing from healthy ex-smoker to healthy smoker, experiencing a fatal or nonfatal MI, stroke, COPD, lung cancer, or entering a severe psychiatric episodic health state (e.g., psychiatric hospitalization and/or suicide attempt), or they may die from another cause.

Each health transition and health state incurs associated treatment/management costs. Associated health costs and risk of disease-related mortality can differ over time since initial episode/diagnosis (41). The Markov cycles will continue until the entire cohort has either died or reached aged 85 years (41). The same model structure will be used to estimate the broader benefits and cost savings of scaling up Quitlink to a larger population cohort of people with SMI.

Existing evidence for transition probabilities for the different disease states and utility weights attached to life lived with those health states used in Hurley et al. (41) and Godfrey et al. (24) will be considered for use in this model, subject to an updated literature search. Smoking relapse rates will be estimated using the large longitudinal Household Income Labour Dynamics in Australia (HILDA) survey data. Specifically, relapse rates of data for people who self-report poor mental-health-related quality of life in the early HILDA waves on the included Short Form-12 item (SF-12) instrument will be analyzed.

The longer term health care system costs incurred by the two intervention arms will comprise actual health care resource usage obtained from the government subsidized MBS and PBS database (which will provide data of up to 4 years for the early study enrolments) and health care cost information from the Australian Institute of Health and Welfare (AIHW)—to estimate costs of acute and ongoing management associated with the stated main model health states. Where there is potential for double counting across the two data sources, conservative inclusion decisions will be made.

Where transition rate, utility weight, and health cost data are available specifically for people living with SMI (and if possible, in Australia), it will be used to populate the model. Given that most of such data are currently unavailable, data from general population studies will be employed, coupled with a discussion on how the likely cost effectiveness of Quitlink may be impacted. All model parameters will be subject to an updated literature search at the end-point of the clinical trial to identify if potentially more suitable model data have become available.

### Uncertainty and Scenario Analyses

All analyses in both the trial-based cost-efficacy and modeled cost-effectiveness evaluations will be subjected to both one-way and probabilistic sensitivity analysis where the impacts of plausible variation in data parameters will be tested, using confidence intervals around for example, utility weights, and health costs associated with different health states. This will provide an understanding of which values or assumptions are associated with the greatest amount of uncertainty. As previously mentioned, by necessity, some model parameters will be populated with data from the general population, rather than specifically people with SMI. Given this, scenario analyses will be conducted to investigate the impact of SMI-related data adjustments, which expert opinion suggests is, *prima facie* appropriate, where there is only poor quality or no data available for a given parameter to test uncertainty. These will include, for example, different transition risks and lower utility scores attached to health states for people with SMI compared with general population data used, as well as uncertainty around treatment costs for the main modeled health states for people with SMI. These analyses will also enable estimates of the probability that Quitlink is cost effective to aid funding decision-makers in light of such model uncertainty.

Results of a number of sensitivity tests will be reported on a cost-effectiveness plane and as acceptability curves. The Australian Government has no explicit threshold for what it considers cost effective; however, there exists implicit evidence that the Pharmaceutical Benefits Advisory Committee view interventions that achieve an incremental cost effectiveness ratio (ICER) of no more than AUD45,000 per additional QALY gained, as cost effective (44). This threshold will be applied for the cost-effective acceptability analysis. Published results will include a discussion of model validity, comparing results with those of other smoking cessation models including those reported in the literature review by (24).

## Discussion

This protocol sets out a plan to assess the cost effectiveness of Quitlink versus usual care *via* both trial-based and modeled economic evaluations. The publication of this protocol has two purposes. First, we aim to inform the research and broader public health communities of the conduct of this economic evaluation alongside the Quitlink trial. Second, we set out the plan for analyses *a priori*, thereby reducing potential biases made from *ad hoc* analytic decisions. Any deviations from this protocol will be described and justified in final analyses. In the event that no significant difference is found for the primary outcome, the described economic evaluation may be undertaken where there is a) significant change in key secondary outcomes (QALYs or number of cigarettes smoked) or b) compelling evidence suggesting the sample lacked power or insufficient follow-up to detect a likely significant difference. While we are setting out to identify and collect the best available data to establish the cost effectiveness of Quitlink, there are a number of potential limitations. The exclusion of so-called second-hand (or passive) smoking effects may result in an underestimation of the true benefits to the health care system and broader society as a result of any observed Quitlink treatment effect. For the trial-based evaluation, there is a risk of recall bias in the respondent surveys, relating to—among other data—health service use, medications used, NRTs, and cigarettes purchased. To minimize this potential bias, actual health and medication use data will be obtained from Australian Government MBS and PBS schemes. Further, in the respondent surveys, the recall period that participants will be asked to consider will be deliberately kept short. A further potential risk to data reliability relates to HRQoL. While the AQoL-8D has been shown to be more sensitive to changes in people’s mental health than other instruments (37), there is a risk that the sample size and duration may be insufficient to identify the expected small changes in mental-health-related quality of life within the trial period.

The *a priori* expectation for benefits to largely occur well beyond the trial period (as has been largely demonstrated for smoking cessation interventions) justifies the decision to model future benefits and costs. However, the model-based analyses also carry a number of potential limitations. For the sake of transparency, a Markov model structure has been proposed that includes only a limited number of the full range of smoking-related health states experienced by current and past smokers (24, 25) concentrating on the health and health system impacts of MI, stroke, COPD, lung cancer, and psychotic-related hospitalizations. While these conditions are responsible for an estimated 80% of the diseases and economic burden associated with smoking morbidity and mortality in the Australian setting, there are other smoking-related health issues which will not be included (41). Should Quitlink be found to be effective, the exclusion of other diseases from the model underestimates the true cost effectiveness of Quitlink. Furthermore, it is anticipated that much of the data for the smoking cessation modeling will come from the general population estimates; such data may not reflect the utilities or health costs or transition risks of people with SMI. An updated literature review for all parameters will be conducted at the end of the trial period to ensure up to date and relevant data is used for all model parameters.

Smoking places significant additional financial burden on people with SMI, a particularly financially vulnerable subpopulation. Any financial implications for people with SMI, seen through changes in cigarette consumption, out of pocket costs of health resource utilization and productivity will be presented, providing valuable information on the equity impacts of Quitlink. This research project will conduct analyses and present results of most relevance to smoking cessation program designers and health-funding decision makers. Quitlink has been designed for and will be trialed in a setting where Quitline and mental health peer workers are established parts of the health sector. The cost-effectiveness findings may not be generalizable to settings where such foundations for Quitlink are not in place.

## Conclusion

The primary aim of this economic evaluation will be to establish the cost effectiveness of Quitlink. This protocol for the economic evaluation sets out *a priori*, the intended analyses to be undertaken. Any deviations from this plan that occur in the final publication of results will be clearly described and justified.

Smoking is a major cause of increased mortality and morbidity, as well as poorer mental-health-related quality of life and financial stress for people with SMI. The cost effectiveness of telephone-based smoking cessation interventions like Quitline (with and without NRT) has been well established in general populations; however, there is little evidence of cost effectiveness for such interventions that specifically target SSMI. Furthermore, evidence suggests that SSMI are less likely to be referred to quitlines. The Quitlink intervention, therefore, aims to bridge this gap. Quitlink utilizes existing mental health peer workforce to link SSMI with a tailored quitline service for SSMI. The research team hypothesizes that the use of these existing workforces and tailored quitline support for SSMI will result in Quitlink being found to be both effective and cost effective and also scalable.

## Ethics Statement

Full ethics clearances have been received for the methods described below from the University of Newcastle (Australia) Human Research Ethics Committee (H-2018-0192) and St Vincent’s Hospital, Melbourne (HREC/18/SVHM/154).

The study is based on the principles of Good Clinical Practice (GCP) according to the Declaration of Helsinki. Potential participants will be given oral and written explanation of the study including the potential risks, their right to withdraw at any time, and the details of data protection and confidentiality and sufficient time to ask questions. A signed consent form will be obtained. Participants will be given the opportunity to agree or decline to being contacted for ancillary studies, without effecting participation in the main trial. A copy of the PICF will be given to the person.

## Author Contributions

RS led the conceptual design and writing of this work. MM made substantial contributions to the conceptual design of the work as well as to the writing of this paper. Coauthors all made substantial contributions to the conceptual design of the methods described in this Economic Evaluation Protocol, and all made important contributions in revising the manuscript critically for important intellectual content. All authors have approved of the final version of the submitted manuscript.

## Funding

This study is supported by an NHMRC Project Grant titled “Quitlink: Accessible smoking cessation support for people living with severe and enduring mental illness” (APP1139125).

## Conflict of Interest Statement

The authors declare that the research was conducted in the absence of any commercial or financial relationships that could be construed as a potential conflict of interest.

The reviewer KD declared a shared affiliation, with no collaboration, with one of the authors, DC, the handling editor.

## Footnotes

1 Reserve Bank of Australia—https://www.rba.gov.au/calculator/annualDecimal.html.

2 Bank of England—https://www.bankofengland.co.uk/monetary-policy/inflation/inflation-calculator
