# Supplementary material for: Protocol for an Economic Evaluation of the Quitlink Randomized Controlled Trial for Accessible Smoking Cessation Support for People With Severe Mental Illness
Source: Front Psychiatry. 2019 Sep 3;10:618. doi: 10.3389/fpsyt.2019.00618 (PMC6735263; doi:10.3389/fpsyt.2019.00618)
Supplement: Supplementary file 1 [file DataSheet_1.docx]

**Online Appendix:**

**Protocol for an economic evaluation of the Quitlink randomised controlled trial for accessible smoking cessation support for people with severe mental illness**

**A1. Introduction**

Presented below are the self-reported Resource Utilisation and Financial Stress questions that will be used in the economic evaluation of the Quitlink intervention. These questions are a subset of the full study assessment questionnaire. Note, these questions are asked at different time-points (Baseline, 2, 5 and 8 months) and the specific wording may differ slightly for some questions to reflect the relevant time-point and time-frame.

**A2. Self-reported service use and out-of-pocket expense related questions**

**A2.1. Cigarette usage**

This data will be incorporated with self-reported (a) quit dates and (b) abstinence periods to estimate out-of-pocket cigarette expenditure.

| 1. How many cigarettes do you smoke per day? |  |
| --- | --- |
| *If participant is a non-daily smoker, instead* *ask*  2. How many cigarettes do you smoke per week? |  |

**A2.2. Products/services to help quit**

| 1. In the last month, have you used any of the following products to help you quit or cut down your tobacco smoking? | | | | | |  |
| --- | --- | --- | --- | --- | --- | --- |
| 1. Please tick all that you have used in the last month. (If none, go to Q4 | 1. About how many packets/cartridges have you used in the last month | | | 1. If yes, have you used this in the last week? | |  |
| 🞎 Patches | ___ packets | | | 🞎 Yes | 🞎 No |  |
| 🞎 Gum | ___ packets | | | 🞎 Yes | 🞎 No |  |
| 🞎 Lozenges | ___ packets | | | 🞎 Yes | 🞎 No |  |
| 🞎 Inhalators | ___ packets | | | 🞎 Yes | 🞎 No |  |
| 🞎 Spray/Mist | ___ packets | | | 🞎 Yes | 🞎 No |  |
| 🞎 Electronic Nicotine Devices (e.g. electronic cigarettes) | ___ cartridges | | | 🞎 Yes | 🞎 No |  |
| 🞎 Champix also called Varenicline or Chantix (prescription medication) | ___ packet(s) | | | 🞎 Yes | 🞎 No |  |
| 🞎 Zyban also called bupropion or Wellbutrin (prescription medication) | ___ packet(s) | | | 🞎 Yes | 🞎 No |  |
| 🞎 Other nicotine replacement products __________ | ___________ | | | 🞎 Yes | 🞎 No |  |
| 🞎 None |  | | |  |  |  |
|  | | | | | | |
| 2. In the last month, have you used any of the following health or other support providers to help you stop or cut down your tobacco smoking? | | | | | | |
| Please tick all that you have used in the last month. | | B. If yes, about how many times did you use this support service or product in the last month? | | | | |
| 🞎 Quitline counselling | | times | | | | |
| 🞎 Individual counselling (excluding Quitline) | | times | | | | |
| 🞎 Acupuncture | | times | | | | |
| 🞎 Hypnotherapy | | times | | | | |
| 🞎 Internet support / online therapy | | times | | | | |
| 🞎 Group counselling program / Group therapy | | times | | | | |
| 🞎 Other, please specify ____________________________________________ | | times | | | | |
| 🞎 None | |  | | | | |
|  | |  | | | | |
| 3. In the last month, have you purchased, downloaded, borrowed or accessed any of the following products to assist quitting (excluding Quitline)? | | | | | | |
|  | | | If yes, how many different products have you purchased? | | | |
| 🞎 Smartphone Self-Help app | | |  | | | |
| 🞎 Self-Help E-books or magazines | | |  | | | |
| 🞎 Self-Help DVDs | | |  | | | |
| 🞎 Other, please specify ____________________________________________ | | |  | | | |
| 🞎 None | | |  | | | |
|  | | | | | |  |

**A2.3. Hospitalisations & other intensive health service use**

| 1. Since you last spoke to the research team/in the last 6 months, have you used any of the following as a patient? If yes, how many times? (If none, go to Q2) | | |
| --- | --- | --- |
| Service type: | No. of times | |
| 🞎 Ambulance | ___ times | |
| 🞎 Emergency Department for a mental health problem | ___ times | |
| 🞎 Emergency Department for a physical health problem (including drug and alcohol related) | ___ times | |
|  | | |
| 2. In the past 6 months, how many times have been admitted for at least one night into the following facilities? Then – thinking of the most recent admission, how many nights did you stay? | | |
| 🞎 General medical hospital for a mental health problem | ___ times | ___ nights |
| 🞎 General medical hospital for a physical health problem (including drug and alcohol related) | ___ times | ___ nights |
| 🞎 Community care unit (CCU) | ___ times | ___ nights |
| 🞎 Preventive and recovery care (PARC) centre | ___ times | ___ nights |
| 🞎 Other (specify) __________________________ | ___ times | ___ nights |

**A2.4. Current medications**The following collected information may be used to supplement Pharmaceutical Benefits Scheme (PBS) data.

| 1. List ALL medication including vitamins, homeopathic or naturopathic remedies, etc. | | | | | |
| --- | --- | --- | --- | --- | --- |
| Medication  (Generic Name) | Dose  & Unit | Freq. | Average dose/ day | | Route |
|  |  |  |  | |  |
| **Frequency:**  01 = Daily  02 = 2 times a day  03 = 3 times a day  04 = 4 times a day  99 = Other, specify (e.g. PRN) | **Route:**  01 = Oral  02 = Subcutaneous  03 = Intramuscular  04 = Intravenous  05 = Rectal | | | 06 = Topical  07 = Respiratory  08 = Sublingual  09 = Transdermal  99 = Other, specify | |

**A2.5. Time off from work and usual duties**

| 1. Since you last spoke to the research team/in the last 3 months, about how many days have you had to take off from any paid work? |
| --- |
| 🞎 ________ days |
| 2. Since you last spoke to the research team/in the last 3 months, about how many days have you had to take off from any UNpaid work, such as study, volunteer work, caring duties, house-keeping etc? |
| 🞎 ________ days |

**A2.6. Financial stress**

| The next few questions will focus on your financial situation and how you feel about your finances. | | | |
| --- | --- | --- | --- |
| 1. In the last month^a^ did any of the following happen to you because of a lack of money? | | | |
|  | **No** | **Yes** | **N/A** |
| a. Could not pay electricity, gas or telephone bills on time | 🞎 | 🞎 | 🞎 |
| b. Could not pay the mortgage or rent on time | 🞎 | 🞎 | 🞎 |
| c. Pawned or sold something | 🞎 | 🞎 | 🞎 |
| d. Went without meals | 🞎 | 🞎 | 🞎 |
| e. Was unable to heat or air condition the home | 🞎 | 🞎 | 🞎 |
| f. Asked for financial help from friends or family | 🞎 | 🞎 | 🞎 |
| g. Asked for help from a welfare or community organisation | 🞎 | 🞎 | 🞎 |
|  | | | |
| 2. In the last month^a^, has there been a time when the money you spent on cigarettes resulted in not having enough money for household essentials such as food? | | | |
| 🞎 No | | | |
| 🞎 Yes | | | |
| 🞎 Don’t know | | | |
|  | | | |

^a^ Note, the timeframe for these questions covers the previous 1 month at Baseline, then 2 months at the 2-month follow-up and 3 months at the 5 and 8-month follow-ups.
